# Integration of primary contact physiotherapists in the emergency department for individuals presenting with minor musculoskeletal disorders: Protocol for an economic evaluation

**DOI:** 10.1371/journal.pone.0277369

**Published:** 2023-09-14

**Authors:** Rose Gagnon, Luc J. Hébert, Jason R. Guertin, Simon Berthelot, François Desmeules, Kadija Perreault

**Affiliations:** 1 Centre Interdisciplinaire de Recherche en Réadaptation et Intégration Sociale, Centre Intégré Universitaire de Santé et de Services Sociaux de la Capitale-Nationale, Quebec, Quebec, Canada; 2 Department of Rehabilitation, Faculty of Medicine, Université Laval, Quebec, Quebec, Canada; 3 Department of Radiology and Nuclear Medicine, Faculty of Medicine, Université Laval, Quebec, Quebec, Canada; 4 Department of Social and Preventive Medicine, Faculty of Medicine, Université Laval, Quebec, Quebec, Canada; 5 Axe Santé des Populations et Pratiques Optimales en Santé, Centre de Recherche du Centre Hospitalier Universitaire de Québec, Université Laval, Quebec, Quebec, Canada; 6 Department of Family Medicine and Emergency Medicine, Faculty of Medicine, Université Laval, Quebec, Quebec, Canada; 7 Centre Hospitalier Universitaire de Québec, Université Laval, Quebec, Quebec, Canada; 8 School of Rehabilitation, Faculty of Medicine, Université de Montréal, Montreal, Quebec, Canada; 9 Orthopaedic Clinical Research Unit, Maisonneuve-Rosemont Hospital Research Centre, Centre Intégré Universitaire de Santé et de Services Sociaux de l’Est-de-l’Île-de-Montréal, Montreal, Quebec, Canada; The University of Sydney, AUSTRALIA

## Abstract

**Objectives:**

1) To compare the average cost of an emergency department (ED) visit for various minor musculoskeletal disorders between two models of care (physiotherapist and ED physician or ED physician alone); 2) To evaluate the incremental cost-effectiveness ratio (ICER) of these two models of care over a 3-month period post-initial visit; and 3) To estimate the ICER of three ED models of care (physiotherapist and ED physician, ED physician alone, physiotherapist alone) over a two-year period.

**Methods:**

Obj.1: The costs incurred by participants in the two groups during their ED visit will be calculated using the Time-Driven Activity-Based Costing (TDABC) method. These costs will be compared using generalized linear models. Obj. 2: The ICER of the two models will be evaluated over three months via a cost-utility analysis that will combine costs and effectiveness data (quality-adjusted life years) using both Health system and Societal perspectives (patient + health system costs). Obj. 3: The 2-year ICER of the three above-mentioned models will be estimated using a mathematical model including a decision tree (0–3 months post-visit) and a Markov model (3–24 months post-visit), also using both Health system and Societal perspectives. Data to answer the three objectives will come from data collected during a randomized clinical trial (n = 78, CHU de Québec)which will be supplemented with data obtained via some of the CHU de Québec administrative databases (nominative data; SIURGE (ED management software), Cristal-Net (patient electronic record), and the ED’s pharmacy transactions directory; administrative data: drug costs repository), the literature, and public cost repositories.

**Conclusion:**

This study will help to determine which model of care is most efficient for the management of individuals who come to the ED with minor musculoskeletal disorders. The increased involvement of various health professionals in the management of patients in the ED paves the way for the development of new avenues of practice and more efficient organization of services.

## Introduction

The emergency department (ED) serves as the main gateway and the preferred resource when primary care services are not available, for example in cases of lack of affiliation with a primary care source or inability to see a physician within a reasonable time frame [1–5]. Although pain conditions for which patients decide to go to the ED are varied, they are oftentimes related to a musculoskeletal disorder (MSKD) [6–8].

According to the World Health Organization, MSKDs are characterized by "pain (often persistent) and limitations in mobility, dexterity and general functioning" [9]. MSKDs can affect joints, bones, muscles, spine and multiple regions of the body [9, 10]. The prevalence of these disorders is reported to be significantly higher in women, older people and people with low socio-economic status [11–15]. When they do not receive timely and appropriate care, people with MSKDs tend to make greater use of health care services and resources [16–22]. MSKDs account for up to 12.6% of a country’s total health care costs each year [15] and this figure is expected to rise with the increase in obesity, physical inactivity and the aging of the population [11, 23]. It is therefore essential to study the costs and clinical effectiveness of interventions aimed at managing MSKDs in order to choose the most efficient ones, including in the ED.

Various models of care have been implemented in the ED and studied in recent years to optimize the management of people presenting with MSKDs. These models of care aim to optimize the flow of patients to and in the ED in three distinct phases: "input" (i.e., flow of patients deciding to come to the ED), "throughput" (i.e., flow of patients while in the ED), and "output" (i.e., flow of patients upon discharge from the ED) [24]. Such models of care include for instance fast-track corridors for patients with minor injuries or rapid assessment teams [25]. Some models include the addition of ED nurse practitioners and a variety of health professionals with a usual or extended scope of practice, such as the primary contact physiotherapist or advanced practice physiotherapist [25].

The addition of primary contact physiotherapists in the ED is an emerging model of care that aims to optimize patient flow while in the ED [25]. Several studies conducted in recent years have shown that this model of care is associated with reduced time waited before receiving care, and reduced length of stay in the ED, as well as fewer unnecessary consultations with various health professionals, and less prescriptions of imaging tests and medication, including opioids, and over-the-counter medication [8, 26–29]. In addition, this model of care was associated with fewer repeat visits to the ED for a similar condition for up to one month after the initial ED visit [29]. Thus, management by a primary contact physiotherapist appears to be associated with decreased service and resource use, both at the ED and up to several weeks later. However, very few studies having investigated primary contact physiotherapist care in the ED have looked at its cost-effectiveness.

Indeed, despite evidence of clinical benefits associated with the presence of a primary contact physiotherapist in the ED (effectiveness), scientific evidence remains rather scarce regarding the cost-effectiveness of this model of care. Two studies conducted in primary care settings (primary care clinic and private clinic) report that primary contact physiotherapist management is associated with a slight increase in health-related quality of life and a decrease in total costs compared to usual management by a family physician [30, 31]. In addition, early physiotherapy management was associated with a decrease in total MSKD-related costs for up to two years after initial management [17, 19, 32]. Two cost-minimization studies conducted in Great Britain looked specifically at the costs associated with the integration of a primary contact physiotherapist in the ED compared to usual management by an emergency physician. According to the study by Richardson et al. (2005, n = 766 patients with non-fracture MSKDs), the presence of a primary contact physiotherapist in the ED results in costs equivalent to usual management (emergency physician) [33]. Similarly, according to McClellan et al. (2013, n = 372 patients >16 years of age with a peripheral MSKD), management by a primary contact physiotherapist results in costs at least as high as usual management (emergency physician) [34]. Nevertheless, in addition to having been conducted exclusively in Great Britain several years ago, these two studies only measured the costs of the two models of care compared and not their effectiveness, the authors assuming that the two models compared were equivalent in terms of clinical effectiveness. These studies are thus not considered to be formal economic evaluation according to current guidelines, but rather a costing exercise, in that a cost-effectiveness analysis accounts for the uncertainty associated with the effects of the interventions being compared [35]. To our knowledge, no other study has examined the cost-effectiveness of primary contact physiotherapy in the ED. Furthermore, no study has assessed whether involving primary contact physiotherapists in the ED have a long-term impact on use of health system services and resources for persons with minor MSKDs. Consequently, further evidence is needed on the efficiency of integrating a primary contact physiotherapist in the ED compared to usual management by an emergency physician.

Therefore, the general objective of this project is to evaluate the efficiency of different models of care for the management of minor MSKDs in the ED. More specifically, the objectives are to:

1. Compare the average costs of an ED consultation and care for various MSKDs, according to two models of care:
  - Usual management by an emergency physician
  - Primary contact physiotherapist management + emergency physician management
2. Evaluate the incremental cost-effectiveness ratio (ICER), from both Health system and Societal perspectives, of these two ED models of care for the management of MSKDs over a three-month period post-initial ED visit.
3. Estimate the ICER between three ED models of care for MSKD management over a two-year period from both Health system and Societal perspectives:
  - Usual management by an emergency physician
  - Primary contact physiotherapist management + emergency physician management
  - Primary contact physiotherapist management alone

## Materials and methods

### Study design and costing approaches

This study is composed of three distinct designs, one per objective. The costing approach used for Objective 1 will be Time-Driven Activity-Based Costing (TDABC), which involves determining the per-minute costs associated with each care process included in a care pathway by multiplying the cost per minute of each care process by its duration. Details on this costing approach and its application to the ED have been described by one of the authors elsewhere [36]. Objective 2 will be achieved through a cost-utility analysis approach in which health care costs at the ED visit and those reported at the 1- and 3-month follow-ups will be compiled and combined with the utility scores obtained at the same measurement times, from both Health system and Societal perspectives. Cost-utility analysis is favored in Canada since it uses a generic outcome measure allowing comparison of the health gains associated with several different interventions, such as different models of care [35].

The ICER between the three ED models of care for the management of MSKDs over a two-year period (Objective 3) will be estimated using a cost-effectiveness analysis via a hybrid mathematical model. This model will consist of a decision tree covering the period from the initial ED visit up to three months post-initial visit, and a Markov model starting three months post-initial ED visit and ending 24 months (two years) after the ED visit. The decision tree provides a simple and clear illustration of a patient’s possible short-term care pathways following a new intervention [35, 37]. In addition to reporting the different interventions used, the decision tree also allows for the inclusion of adverse events following the initial intervention, such as a new ED visit for the same condition, and for repeating an intervention over time as needed (e.g., new visit in the ED a few days after the initial visit and then a new visit two months later for the same condition) [35, 37]. It also permits to determine the proportion of disability associated with each of the three ED models of care.

Several considerations guided the choice of the time horizon for the Markov model. First of all, to be considered chronic, a musculoskeletal disorder must be present for at least three months [38]. Moreover, approximately 30% of people presenting with MSKDs report pain and functional disability lasting more than 12 months after the onset of their condition. Furthermore, studies on MSKD care in primary care or the ED have had follow-up periods ranging from six to 24 months (e.g. [26, 39–42]). Thus, the Markov model will cover a 24-month period. It will include two-week cycles in order to capture the clinical evolution of the patients included.

### Population of interest

Inclusion and exclusion criteria for the population of interest are described in Box 1. The population targeted covers persons with a peripheral or spinal MSKD, a P3, P4 or P5 triage category at the ED (Canadian Triage and Acuity Scale [43]), and that are aged between 18 and 80. Having a major MSKD, red flag or associated unstable condition are criteria for exclusion.

**Box 1:**
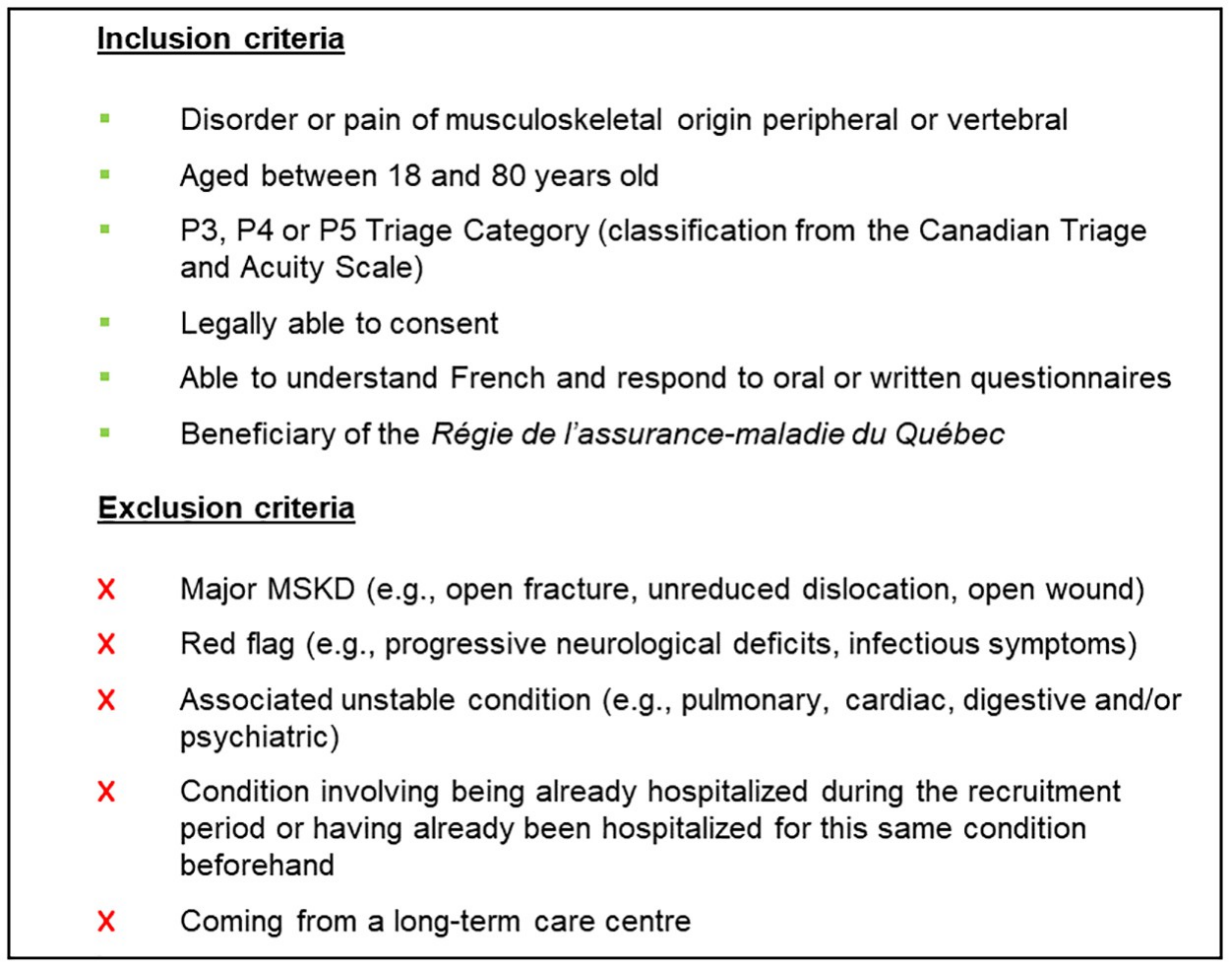
Inclusion and exclusion criteria for the population of interest.

### Data collection

Objectives 1, 2 and 3 will be achieved in part using data collected through a two-arm pilot pragmatic randomized clinical trial (RCT) conducted in the ED of the CHUL, one of the five sites of the CHU de Québec—Université Laval (UL) (Quebec City, Canada) from September 2018 to March 2019 (n = 78). This trial aimed to compare the effects of management by a primary contact physiotherapist to usual care provided by an emergency physician for persons presenting with a minor MSKD on their clinical course (pain and pain interference) and the use of resources at ED discharge and after 1 and 3 months post-visit [29]. Two groups of participants were compared: one group managed by a primary contact physiotherapist and an emergency physician and one group managed by an emergency physician alone.

Data were collected at the initial ED visit and at the one- and three-month post-visit follow-ups. While more details of the data collection procedures can be found in our previous paper [29], any person presenting to the ED who met the inclusion and exclusion criteria was seen by a member of the research team who confirmed eligibility, obtained informed consent, and ensured completion of baseline questionnaires. The participant was then randomized to either study group: primary contact physiotherapist + emergency physician management or usual management by the emergency physician alone. After the ED visit was completed, participants were contacted at 1 and 3 months either by phone or email to complete post-visit follow-ups. The supplementary data needed to meet the three Objectives will come from some of the CHU de Québec—UL administrative databases (nominative data: SIURGE (ED management software), Cristal-Net (patient electronic record), and the CHUL’s ED pharmacy transactions directory; administrative data: prescription and over-the-counter drug costs repository), the scientific literature, and public cost repositories (e.g., salaries of general practitioners and specialists, see https://www.msss.gouv.qc.ca/inc/documents/ministere/acces_info/seance-publique/etude-credits-2018-2019/Reponses-aux-questions-generales-et-particulieres-RAMQ.pdf; laboratory analysis costs, see https://publications.msss.gouv.qc.ca/msss/fichiers/2017/17-922-05W.pdf).

Of note, the study population for the model of care consisting of primary contact physiotherapist management and discharge from the ED was not observed at all during the pilot pragmatic RCT. Consequently, this model of care will have to be modeled, and therefore cannot be studied in the context of Objectives 1 and 2, which are based on RCT data. All the parameters needed to represent this care model within Objective 3 (probabilities, costs, measures of effectiveness) will be taken from a literature review, an approach regularly used in economic evaluation [44]. However, the studies from which the metrics will be derived will need to have a sample that meets the same inclusion criteria as those presented in Box 1. Data extracted from the literature will be validated with members of the research team and with experts in the field of emergency medicine, MSKDs and rehabilitation if necessary during the construction of the hybrid model [35].

### Study outcomes

Primary outcomes used to measure the average cost of an ED visit (Objective 1) will be the costs of care processes and the time associated with each care process. This method of costing is routinely used by some members of the research team [36, 45, 46]. The costs related to ED management (medical and non-medical staff, imaging, medication, consumables, maintenance, etc.) were obtained via a formal request made by a member of the research team to the CHU de Québec–UL Finance Department. The time associated with each care process was calculated by a member of the research team using estimates provided by the CHUL medical and non-medical staff that were validated during an observation period in the ED [36, 45]. Uncertainty in measured times will allow the variability of ED costs to be considered, and to derive a distribution of possible costs at baseline for each participant.

As part of the cost-utility analysis (Objective 2) and hybrid mathematical model (Objective 3), the efficiency of the ED models of care will be assessed using an incremental cost-effectiveness ratio (ICER). The resulting ICER will be reported in terms of incremental cost per quality-adjusted life years (QALY) gained, between the models of care. The follow-up costs of each participant recorded via the self-administered follow-up questionnaires completed at 1 and 3 months during the pilot pragmatic RCT will be added to the distribution of ED visit costs for each individual obtained under Objective 1. Once all costs have been added, an average will be calculated to obtain an average cost per participant at 3 months, for each model of care (Objective 2 & 3 –decision tree). The questionnaires provided data on resources used by each participant during follow-up such as ED re-visits for the same condition, number of consultations with other health professionals in the public and private sector, imaging tests used, etc. The costs of all resources used in the public healthcare system will be drawn from public cost repositories data from the *Régie de l’assurance-maladie du Québec* (RAMQ) (costs related to the emergency physician and other physicians consulted, drugs, laboratory analysis, imaging tests) [47]. Hourly rates for the primary contact physiotherapist and other health professionals consulted (e.g., massage therapist, chiropractor, osteopath) will be derived from a search of the grey literature and will be based on rates in effect within the private healthcare system, since the majority of these professionals work within this system [47]. Costs related to the use of technical aids (e.g., cane, crutches, walker) will also come from a grey literature search. It should be noted that the salary data available from human resources for all healthcare professionals (physicians and allied health professionals) considered will not allow for variability in hourly costs. The only source of variability considered for the purpose of these analyses will be that related to the time required for ED processes. Utility scores were obtained at the initial visit [48] and at 1 and 3 months using the EQ-5D-5L, a generic standardized questionnaire designed to measure health status in an economic and clinical evaluation [49]. The EQ-5D-5L has been found to be reliable, valid, and sensitive to change [50, 51]. The difference in utility scores between the 3-month follow-up and baseline will be calculated for each participant using area-under-the-curve analyses. Once the difference in utility scores will be calculated for each participant, the differences will be averaged to obtain the average gain or loss in utility scores per ED care model. The mean gain or loss in utility scores will then be transformed into QALYs. The efficiency values and the costs from 3 to 24 months required to run the Markov model (Objective 3) for the three models of care will be taken from the literature.

### Data analysis and interpretation of results

**Fig 1:**
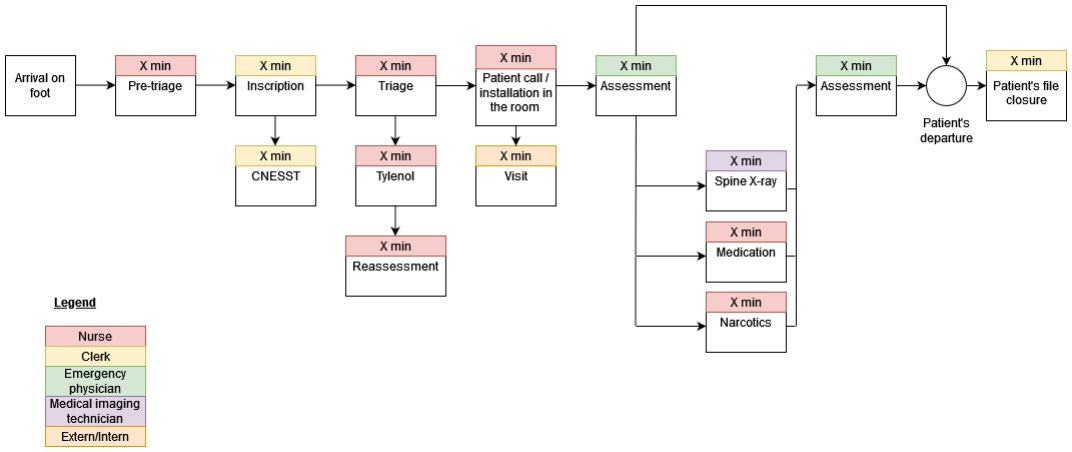
Mapping of a hypothetical care pathway in the ED using the Time-Driven Activity-Based Costing.

As part of Objective 1, a mapping of the care pathways encountered will be completed for each type of MSKD encountered in our study population (i.e., low back pain, neck pain, upper limb, lower limb) (Fig 1). The unit cost of each of the resources, consumables and indirect costs required in each process of care of the care pathway will be calculated and multiplied by the duration of each process to obtain the cost related to each process of care present in the care pathway. The costs associated with each process will be summed to obtain the total cost of the ED care pathway specific to each MSKD and each model of care (i.e., emergency physician or primary contact physiotherapist and emergency physician management). A generalized linear model with a Gamma distribution and log link will be used to test whether there is a significant difference in the costs of managing equivalent MSKDs between the two models of care [52, 53].

**Fig 2:**
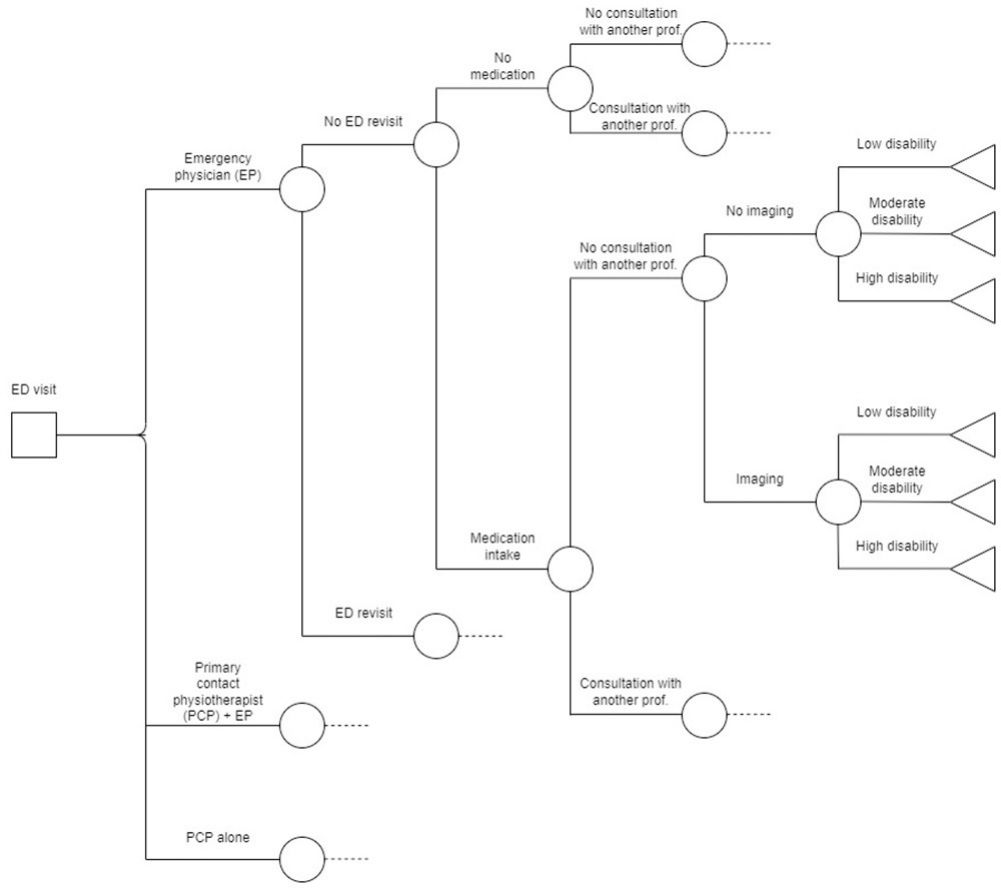
Hypothetical decision tree covering the period from ED visit to three months post initial ED visit.

**Fig 3:**
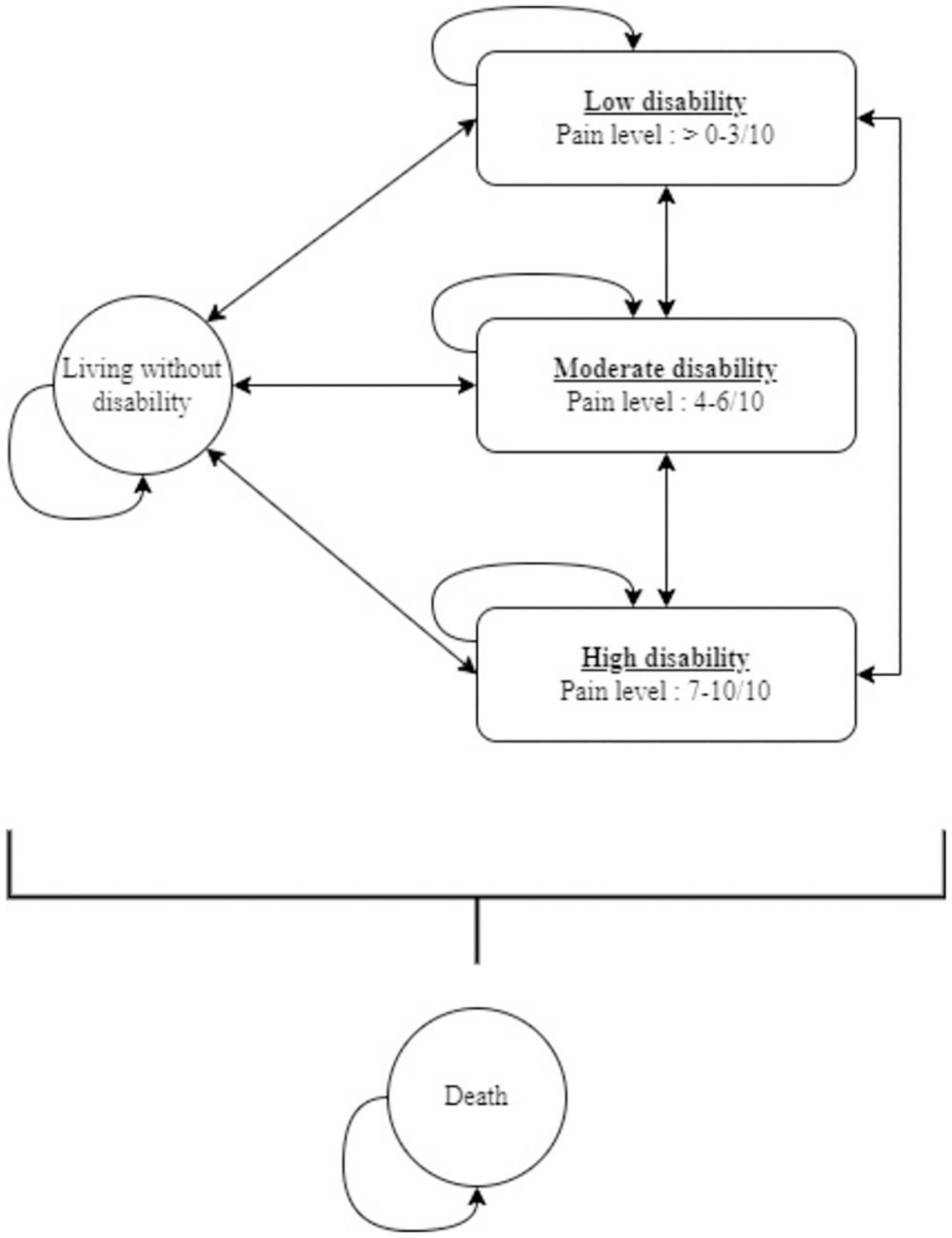
Projected Markov model covering the period from three to 24 months post initial ED visit.

The decision tree (Objective 3) will be created to reflect the results of the RCT as closely as possible and will therefore include all possible interventions and services used by a participant following the initial visit to the ED for each model of care considered (Fig 2). The pruning of each terminal node containing less than 5% of the participants will be determined based on discussions with a panel of experts. The conditional probability of ending up in each of the remaining terminal nodes of the decision tree will be used to calculate the proportion of disability associated with each care model (Fig 2). The disability proportions obtained for each model of care will be used to determine the number of individuals in each state at entry in the Markov model (Fig 3). The Markov model will then be used to calculate the long-term costs and effectiveness over 2 years of each of the model of care based on the level of disability estimated in the decision tree [35, 37].

Both the cost-utility analysis (Objective 2) and the hybrid mathematical model (Objective 3) will be conducted from a Health system and a Societal perspective. Results obtained via the EQ-5D-5L at 1 and 3 months (Objective 2 & 3 –decision tree) will be converted to utility scores using the Canadian conversion algorithm developed by Xie et al. [54]. As the 3-month retention rate for the pilot pragmatic trial was 80% [29], some participants’ data are missing (service and resource use, costs, utility scores). Missing data will be imputed using the Missing not at random (MNAR) multiple imputation method [55]. Patient characteristics believed to have influenced loss to follow-up at 1 and 3 months (e.g., socioeconomic status; level of pain and level of catastrophizing at the ED visit) will be examined to identify if they are indeed predictors of loss to follow-up. Characteristics shown to influence follow-up will be used within multiple imputations and within sensitivity analyses regarding these variables. Uncertainty in cost and effectiveness measures for the cost-utility analysis (Objective 2) will be obtained using non-parametric bootstrap resampling with replacement. Uncertainty in the hybrid model parameters (probabilities, costs, and efficiency) (Objective 3) will be obtained via a probabilistic sensitivity analysis performed using a Monte Carlo simulation. Three scenarios will be considered in the probabilistic sensitivity analyses. The first will assume that all participants with missing follow-up data in the control group will have utility scores at 3 months corresponding to those of the 95th percentile, while all those missing in the intervention group will have scores corresponding to those of the 5th percentile. For the second scenario, we will re-analyze all parameters included in the model, excluding outliers. Finally, in the third and last scenario, the different states of the Markov Model will be based not on the level of pain, but on the level of interference of pain on daily activities. Both uncertainties will be represented visually using a cost-effectiveness diagram, cost-effectiveness acceptability curve, and cost-effectiveness acceptability frontier [35]. As there are significant differences between men and women in the strategies used to cope with pain, and the use of health system services and resources during a painful episode (e.g., [56, 57]), subgroup analyses of men and women will be performed for both objectives (p < .05). Subgroup analyses will also be performed according to MSKD category (spine, upper extremity, and lower extremity) for both objectives (p < .05). All the statistical analyses carried out as part of this project and their detailed reporting in scientific publications will adhere to the CHEERS 2022 standard [58].

### Ethical considerations and data management

This project and all of its components (conduct of the RCT, three-month participants’ follow-up, access to nominative and administrative databases) were approved by the CHU de Québec–UL’s Ethical review board (approval number: MP-20-2019-4307). The randomized clinical trial was also registered with the US National Institutes of Health (#NCT04009369). Each participant signed a written consent form prior to participation. All data collected will be kept in a secure repository and destroyed thereafter. All members of the research team signed a confidentiality agreement.

## Discussion

The overall aim of this project is to evaluate the costs of different models of care for the management of MSKDs in the ED. This will be achieved through three specific objectives: 1) to compare the average costs of an ED consultation and care for various MSKDs; 2) to evaluate the incremental cost-effectiveness ratio (ICER) of two ED models of care for the management of MSKDs over a three-month period post-initial ED visit; and 3) to estimate the ICER between ED models of care for the management of MSKDs over a two-year period.

Until now, there has been no formal economic evaluation of the inclusion of a primary contact physiotherapist in the ED compared with usual practice (emergency physician). The only studies that have been done on the subject have assumed that the effectiveness of the primary contact physiotherapist’s management in the ED is equivalent to that of usual care by the emergency physician. However, several studies have reported that primary contact physiotherapist management can reduce the use of services and resources during the ED stay [8, 26–29, 59]. This research project will fill an important need in the literature by providing an in-depth analysis of the costs and efficiency of the considered models of care. Indeed, this project will help identify the most efficient ED model of care. These models of care also have the potential to improve the quality of services offered to people with MSKDs, their clinical evolution and their quality of life. The increased use of various health professionals in the management of patients in contexts such as the ED can pave the way for the development of new avenues of practice and potentially more efficient organization of services that will benefit the population.

This study is associated with some potential limitations. First of all, the data needed to carry out Objectives 1 and 2 will partly be based on results from a pilot pragmatic RCT. Therefore, the results obtained should be interpreted with caution. The small sample size (n = 78) could possibly limit analyses on the number of plausible branches in the final decision tree as well as the amount of subgroup analysis that will be performed. In addition, although high, the retention rate at the 3-month follow-up of the RCT was 80% [29], which implies that some data related to the use of services and resources, costs and health-related quality of life will be missing. However, this limitation will be mitigated using multiple imputation methods [55]. Sensitivity analyses will also be performed to assess the robustness of the results obtained. Finally, it may be difficult to obtain some of the data on medium- and long-term costs and measures of effectiveness for the ED models of care studied in Objective 3 from the scientific literature. Nevertheless, estimates can be obtained by soliciting the opinions of experts in the fields of MSKD management and emergency medicine, as this method is regularly used in modeling [35].

As for knowledge translation, following the project, formal presentations will be made to all key stakeholders at the CHU (emergency physicians, physiotherapists, nurses, orderlies, patient representatives and administrators) on site or remotely to present the results of the study and discuss lessons learned and future avenues. The results of this project will also be shared with provincial stakeholders (professional associations, patient associations and governments). They will also be disseminated at national and international scientific conferences on economics, health services organization and emergency services. Four manuscripts will be published in peer-reviewed journals. If successful, this project will help guide economic evaluations for a large-scale, multi-center trial aiming to improve the management of people presenting with a MKSD in the ED.

## References

[1] Roberge D, Larouche D, Pineault R. L’URGENCE HOSPITALIÈRE: UN SUBSTITUT À LA PREMIÈRE LIGNE? 2007; 12.

[2] Carret MLV, Fassa AG, Kawachi I. Demand for emergency health service: factors associated with inappropriate use. BMC Health Serv Res. 2007;7: 131. doi: 10.1186/1472-6963-7-131 17705873 PMC2034385

[3] Carret MLV, Fassa ACG, Domingues MR. Inappropriate use of emergency services: a systematic review of prevalence and associated factors. Cad Saude Publica. 2009;25: 7–28. doi: 10.1590/s0102-311x2009000100002 19180283

[4] Kraaijvanger N, Rijpsma D, van Leeuwen H, Edwards M. Self-referrals in the emergency department: reasons why patients attend the emergency department without consulting a general practitioner first—a questionnaire study. Int J Emerg Med. 2015;8: 46. doi: 10.1186/s12245-015-0096-x 26644131 PMC4671987

[5] Uscher-Pines L, Pines J, Kellermann A, Gillen E, Mehrotra A. Emergency department visits for nonurgent conditions: systematic literature review. Am J Manag Care. 2013;19: 47–59. 23379744 PMC4156292

[6] Idil H, Kilic TY, Toker İ, Dura Turan K, Yesilaras M. Non-urgent adult patients in the emergency department: Causes and patient characteristics. Turk J Emerg Med. 2018;18: 71–74. doi: 10.1016/j.tjem.2017.10.002 29922734 PMC6005911

[7] Fact Sheets | BMUS: The Burden of Musculoskeletal Diseases in the United States. 2013 [cited 10 Jan 2020]. https://www.boneandjointburden.org/fact-sheets

[8] Bird S, Thompson C, Williams KE. Primary contact physiotherapy services reduce waiting and treatment times for patients presenting with musculoskeletal conditions in Australian emergency departments: an observational study. Journal of Physiotherapy. 2016;62: 209–214. doi: 10.1016/j.jphys.2016.08.005 27637771

[9] World Health Organization (WHO). Musculoskeletal conditions. [cited 15 Feb 2021]. https://www.who.int/news-room/fact-sheets/detail/musculoskeletal-conditions

[10] Ledoux E, Denis D. Enquête québécoise sur des conditions de travail, d’emploi et de santé et de sécurité du travail (EQCOTESST). Perspectives interdisciplinaires sur le travail et la santé. 2011 [cited 13 Jun 2019].

[11] Cieza A, Causey K, Kamenov K, Hanson SW, Chatterji S, Vos T. Global estimates of the need for rehabilitation based on the Global Burden of Disease study 2019: a systematic analysis for the Global Burden of Disease Study 2019. Lancet. 2020;396: 2006–2017. doi: 10.1016/S0140-6736(20)32340-0 33275908 PMC7811204

[12] Wijnhoven HAH, de Vet HCW, Picavet HSJ. Prevalence of Musculoskeletal Disorders Is Systematically Higher in Women Than in Men. The Clinical Journal of Pain. 2006;22: 717–724. doi: 10.1097/01.ajp.0000210912.95664.53 16988568

[13] Treaster DE, Burr D. Gender differences in prevalence of upper extremity musculoskeletal disorders. Ergonomics. 2004;47: 495–526. doi: 10.1080/00140130310001638171 15204301

[14] Urwin M, Symmons D, Allison T, Brammah T, Busby H, Roxby M, et al. Estimating the burden of musculoskeletal disorders in the community: the comparative prevalence of symptoms at different anatomical sites, and the relation to social deprivation. Annals of the Rheumatic Diseases. 1998;57: 649–655. doi: 10.1136/ard.57.11.649 9924205 PMC1752494

[15] March L, Smith EUR, Hoy DG, Cross MJ, Sanchez-Riera L, Blyth F, et al. Burden of disability due to musculoskeletal (MSK) disorders. Best Practice & Research Clinical Rheumatology. 2014;28: 353–366. doi: 10.1016/j.berh.2014.08.002 25481420

[16] Horn ME, Brennan GP, George SZ, Harman JS, Bishop MD. A value proposition for early physical therapist management of neck pain: a retrospective cohort analysis. BMC Health Serv Res. 2016;16. doi: 10.1186/s12913-016-1504-5 27405318 PMC4942887

[17] Childs JD, Fritz JM, Wu SS, Flynn TW, Wainner RS, Robertson EK, et al. Implications of early and guideline adherent physical therapy for low back pain on utilization and costs. BMC Health Serv Res. 2015;15: 150. doi: 10.1186/s12913-015-0830-3 25880898 PMC4393575

[18] Ehrmann-Feldman D, Rossignol M, Abenhaim L, Gobeille D. Physician Referral to Physical Therapy in a Cohort of Workers Compensated for Low Back Pain. Phys Ther. 1996;76: 150–156. doi: 10.1093/ptj/76.2.150 8592718

[19] Fritz JM, Childs JD, Wainner RS, Flynn TW. Primary Care Referral of Patients With Low Back Pain to Physical Therapy: Impact on Future Health Care Utilization and Costs. Spine. 2012;37: 2114–2121. doi: 10.1097/BRS.0b013e31825d32f5 22614792

[20] Gellhorn AC, Chan L, Martin B, Friedly J. Management Patterns in Acute Low Back Pain: the Role of Physical Therapy. Spine (Phila Pa 1976). 2012;37: 775–782. doi: 10.1097/BRS.0b013e3181d79a09 21099735 PMC3062937

[21] Kucera KL, Lipscomb HJ, Silverstein B. Medical care surrounding work-related back injury claims among Washington State Union Carpenters, 1989–2003. Work. 2011;39: 321–330. doi: 10.3233/WOR-2011-1180 21709368

[22] Zigenfus GC, Yin J, Giang GM, Fogarty WT. Effectiveness of Early Physical Therapy in the Treatment of Acute Low Back Musculoskeletal Disorders. Journal of Occupational and Environmental Medicine. 2000;42: 35. doi: 10.1097/00043764-200001000-00010 10652686

[23] Storheim K, Zwart J-A. Musculoskeletal disorders and the Global Burden of Disease study. Annals of the Rheumatic Diseases. 2014;73: 949–950. doi: 10.1136/annrheumdis-2014-205327 24790065

[24] Asplin BR, Magid DJ, Rhodes KV, Solberg LI, Lurie N, Camargo CA. A conceptual model of emergency department crowding. Ann Emerg Med. 2003;42: 173–180. doi: 10.1067/mem.2003.302 12883504

[25] Wylie K, Crilly J, Toloo GS, FitzGerald G, Burke J, Williams G, et al. Review article: Emergency department models of care in the context of care quality and cost: a systematic review. Emerg Med Australas. 2015;27: 95–101. doi: 10.1111/1742-6723.12367 25752589

[26] de Gruchy A, Granger C, Gorelik A. Physical therapists as primary practitioners in the emergency department: six-month prospective practice analysis. Physical therapy. 2015;95: 1207. doi: 10.2522/ptj.20130552 25929528

[27] Heywood JW. Specialist physiotherapists in orthopaedic triage-the results of a military spinal triage clinic. Journal of the Royal Army Medical Corps. 2005;151: 152–156. doi: 10.1136/jramc-151-03-04 16440957

[28] Sohil P, Hao PY, Mark L. Potential impact of early physiotherapy in the emergency department for non-traumatic neck and back pain. World J Emerg Med. 2017;8: 110–115. doi: 10.5847/wjem.j.1920-8642.2017.02.005 28458754 PMC5409230

[29] Gagnon R, Perreault K, Berthelot S, Matifat E, Desmeules F, Achou B, et al. Direct-access physiotherapy to help manage patients with musculoskeletal disorders in an emergency department: Results of a randomized controlled trial. Academic Emergency Medicine. 2021;28: 848–858. doi: 10.1111/acem.14237 33617696

[30] Bornhöft L, Thorn J, Svensson M, Nordeman L, Eggertsen R, Larsson MEH. More cost-effective management of patients with musculoskeletal disorders in primary care after direct triaging to physiotherapists for initial assessment compared to initial general practitioner assessment. BMC Musculoskeletal Disorders. 2019;20. doi: 10.1186/s12891-019-2553-9 31043169 PMC6495522

[31] Denninger TR, Cook CE, Chapman CG, McHenry T, Thigpen CA. The Influence of Patient Choice of First Provider on Costs and Outcomes: Analysis From a Physical Therapy Patient Registry. J Orthop Sports Phys Ther. 2017;48: 63–71. doi: 10.2519/jospt.2018.7423 29073842

[32] Magel J, Kim J, Fritz JM, Freburger JK. Time Between an Emergency Department Visit and Initiation of Physical Therapist Intervention: Health Care Utilization and Costs. Physical Therapy. 2020;100: 1782–1792. doi: 10.1093/ptj/pzaa100 32478851 PMC7530572

[33] Richardson B, Shepstone L, Poland F, Mugford M, Finlayson B, Clemence N. Randomised controlled trial and cost consequences study comparing initial physiotherapy assessment and management with routine practice for selected patients in an accident and emergency department of an acute hospital. Emergency Medicine Journal. 2005;22: 87–92. doi: 10.1136/emj.2003.012294 15662054 PMC1726666

[34] McClellan CM, Cramp F, Powell J, Benger JR. A randomised trial comparing the cost effectiveness of different emergency department healthcare professionals in soft tissue injury management. BMJ Open. 2013;3: e001116. doi: 10.1136/bmjopen-2012-001116 23293239 PMC3549250

[35] Lignes directrices de l’évaluation économique des technologies de la santé au Canada. Ottawa: ACMTS; 2017 Mar p. 80 pages. Report No.: 4e édition. https://www.cadth.ca/fr/a-propos-de-acmts/comment-nous-procedons/lignes-directrices-en-matiere-de-methodologie/lignes-directrices-de-evaluation-economique

[36] Berthelot S, Mallet M, Blais S, Moore L, Guertin JR, Boulet J, et al. Adaptation of time‐driven activity‐based costing to the evaluation of the efficiency of ambulatory care provided in the emergency department. J Am Coll Emerg Physicians Open. 2022;3: e12778. doi: 10.1002/emp2.12778 35865131 PMC9292471

[37] Drummond MF, Sculpher MJ, Claxton K, Stoddart GL, Torrance GW. Methods for the Economic Evaluation of Health Care Programmes. Oxford University Press; 2015.

[38] Kovačević I, Kogler VM, Turković TM, Dunkić LF, Ivanec Ž, Petek D. Self-care of chronic musculoskeletal pain–experiences and attitudes of patients and health care providers. BMC Musculoskelet Disord. 2018;19: 1–10. doi: 10.1186/s12891-018-1997-7 29514616 PMC5842573

[39] Caffrey A, Smart KM, FitzGerald O. Physiotherapist-Led Triage at a Rheumatology-Based Musculoskeletal Assessment Clinic: an 18-Month Service Evaluation of Activity and Outcomes. ACR Open Rheumatol. 2019;1: 213–218. doi: 10.1002/acr2.1022 31777797 PMC6858023

[40] Downie F, McRitchie C, Monteith W, Turner H. Physiotherapist as an alternative to a GP for musculoskeletal conditions: a 2-year service evaluation of UK primary care data. Br J Gen Pract. 2019;69: e314–e320. doi: 10.3399/bjgp19X702245 30962224 PMC6478452

[41] Lau PM-Y, Chow DH-K, Pope MH. Early physiotherapy intervention in an Accident and Emergency Department reduces pain and improves satisfaction for patients with acute low back pain: a randomised trial. Australian Journal of Physiotherapy. 2008;54: 243–249. doi: 10.1016/s0004-9514(08)70003-5 19025504

[42] Bornhöft L, Larsson ME, Nordeman L, Eggertsen R, Thorn J. Health effects of direct triaging to physiotherapists in primary care for patients with musculoskeletal disorders: a pragmatic randomized controlled trial. Therapeutic Advances in Musculoskeletal Disease. 2019;11: 1759720X1982750. doi: 10.1177/1759720X19827504 30800175 PMC6378424

[43] Beveridge R, Clarke B, Janes L, Savage N, Thompson J, Dodd G, et al. L’échelle canadienne de triage & de gravité pour les départements d’urgence Guide d’implantation. Can J Emerg Med. 1999;1.

[44] O’brien B. Economic Evaluation of Pharmaceuticals: Frankenstein’s Monster or Vampire of Trials? Medical Care. 1996;34: DS99–DS108. 8969318

[45] Berthelot S, Mallet M, Baril L, Dupont P, Bissonnette L, Stelfox H, et al. P017: A time-driven activity-based costing method to estimate health care costs in the emergency department. Canadian Journal of Emergency Medicine. 2017;19: S83–S83. doi: 10.1017/cem.2017.219

[46] Berthelot S, Breton M, Guertin JR, Archambault PM, Berger Pelletier E, Blouin D, et al. A Value-Based Comparison of the Management of Ambulatory Respiratory Diseases in Walk-in Clinics, Primary Care Practices, and Emergency Departments: Protocol for a Multicenter Prospective Cohort Study. JMIR Res Protoc. 2021;10: e25619. 33616548 10.2196/25619 PMC7939947

[47] Jacobs P, Budden A, Lee KM. Guidance document for the costing of health care resources in the Canadian setting. Ottawa: CADTH; 2016 Mar p. 50. Report No.: 2nd edition. https://www.cadth.ca/sites/default/files/pdf/CP0009_CADTHCostingGuidance.pdf

[48] Gagnon R, Perreault K, Guertin JR, Berthelot S, Achou B, Hébert LJ. Health-Related Quality of Life of Patients Presenting to the Emergency Department with a Musculoskeletal Disorder. CEOR. 2022;14: 91–103. doi: 10.2147/CEOR.S348138 35221700 PMC8865860

[49] EQ-5D User Guides–EQ-5D. [cited 18 Sep 2019]. https://euroqol.org/publications/user-guides/

[50] Bilbao A, García-Pérez L, Arenaza JC, García I, Ariza-Cardiel G, Trujillo-Martín E, et al. Psychometric properties of the EQ-5D-5L in patients with hip or knee osteoarthritis: reliability, validity and responsiveness. Qual Life Res. 2018;27: 2897–2908. doi: 10.1007/s11136-018-1929-x 29978346

[51] Conner-Spady BL, Marshall DA, Bohm E, Dunbar MJ, Loucks L, Al Khudairy A, et al. Reliability and validity of the EQ-5D-5L compared to the EQ-5D-3L in patients with osteoarthritis referred for hip and knee replacement. Qual Life Res. 2015;24: 1775–1784. doi: 10.1007/s11136-014-0910-6 25555837

[52] Manning WG, Mullahy J. Estimating log models: to transform or not to transform?ଝ. Journal of Health Economics. 2001; 34. 10.1016/s0167-6296(01)00086-8 11469231

[53] Thompson SG, Nixon RM. How Sensitive Are Cost-Effectiveness Analyses to Choice of Parametric Distributions? Med Decis Making. 2005;25: 416–423. doi: 10.1177/0272989X05276862 16061893

[54] Xie F, Pullenayegum E, Gaebel K, Bansback N, Bryan S, Ohinmaa A, et al. A Time Trade-off-derived Value Set of the EQ-5D-5L for Canada. Med Care. 2016;54: 98–105. doi: 10.1097/MLR.0000000000000447 26492214 PMC4674140

[55] RUBIN DB. Inference and missing data. Biometrika. 1976;63: 581–592. doi: 10.1093/biomet/63.3.581

[56] Fullerton EF, Doyle HH, Murphy AZ. Impact of sex on pain and opioid analgesia: a review. Curr Opin Behav Sci. 2018;23: 183–190. doi: 10.1016/j.cobeha.2018.08.001 30906823 PMC6428208

[57] Mills SEE, Nicolson KP, Smith BH. Chronic pain: a review of its epidemiology and associated factors in population-based studies. Br J Anaesth. 2019;123: e273–e283. doi: 10.1016/j.bja.2019.03.023 31079836 PMC6676152

[58] Husereau D, Drummond M, Augustovski F, de Bekker-Grob E, Briggs AH, Carswell C, et al. Consolidated Health Economic Evaluation Reporting Standards 2022 (CHEERS 2022) Statement: Updated Reporting Guidance for Health Economic Evaluations. Appl Health Econ Health Policy. 2022 [cited 11 Jan 2022]. doi: 10.1007/s40258-021-00704-x 35015207 PMC8847248

[59] Matifat E, Méquignon M, Cunningham C, Blake C, Fennelly O, Desmeules F. Benefits of Musculoskeletal Physical Therapy in Emergency Departments: A Systematic Review. Phys Ther. 2019;99: 1150–1166. doi: 10.1093/ptj/pzz082 31505674

